# Selective Connexin43 Inhibition Prevents Isoproterenol-Induced Arrhythmias and Lethality in Muscular Dystrophy Mice

**DOI:** 10.1038/srep13490

**Published:** 2015-08-27

**Authors:** J. Patrick Gonzalez, Jayalakshmi Ramachandran, Lai-Hua Xie, Jorge E. Contreras, Diego Fraidenraich

**Affiliations:** 1 Department of Cell Biology and Molecular Medicine, Rutgers Biomedical and Health Sciences, New Jersey Medical School, Newark, NJ USA; 2 Department of Pharmacology and Physiology, Rutgers Biomedical and Health Sciences, New Jersey Medical School, Newark, NJ USA

## Abstract

Duchenne muscular dystrophy (DMD) is caused by an X-linked mutation that leads to the absence of dystrophin, resulting in life-threatening arrhythmogenesis and associated heart failure. We targeted the gap junction protein connexin43 (Cx43) responsible for maintaining cardiac conduction. In mild *mdx* and severe *mdx:utr* mouse models of DMD, and human DMD tissues, Cx43 was found to be pathologically mislocalized to lateral sides of cardiomyocytes. In addition, overall Cx43 protein levels were markedly increased in mouse and human DMD heart tissues examined. Electrocardiography on isoproterenol challenged mice showed that both models developed arrhythmias and died within 24 hours, while wild-type mice were free of pathology. Administering peptide mimetics to inhibit lateralized Cx43 function prior to challenge protected *mdx* mice from arrhythmogenesis and death, while *mdx:utr* mice displayed markedly improved ECG scores. These findings suggest that Cx43 lateralization contributes significantly to DMD arrhythmogenesis and that selective inhibition may provide substantial benefit.

Duchenne muscular dystrophy (DMD) is the most common and most severe form of muscular dystrophy, affecting nearly 1 in every 3500 newborn males^1^. The disease is characterized by the loss of the critical protein dystrophin, normally responsible for the stabilization of the myocyte sarcolemma through the formation of the dystrophin glycoprotein complex (DGC)^2^. In its absence, cardiac and skeletal muscles are highly susceptible to stress induced damage, especially through adrenergic stimuli such as exercise^3^,^4^,^5^,^6^. Patients with DMD exhibit progressive muscle degeneration and inflammation, leading to the loss of ambulation by the early teens and death in the twenties^7^,^8^. While skeletal, and especially respiratory, muscles have traditionally been targeted therapeutically, heart failure has increasingly become one of the most prevalent causes of death in DMD^9^,^10^,^11^. Recent findings have shown that death of DMD patients attributable to cardiac origins has increased from 8% to 44% between 1970 and 2011, largely due to the use of ventilators in therapy to account for respiratory muscle degradation^12^. Although skeletal and cardiac muscles both require dystrophin for proper function, its role in the heart has remained poorly understood. As a result, cardiac intervention is limited to general heart failure treatments, including β-blockers and ACE inhibitors, due to the inability to successfully identify and target a specific underlying mechanism^13^,^14^,^15^.

While a majority of those with DMD do not display overt cardiac histopathology until closer to adulthood, cardiac arrhythmias have been found to develop in adolescent and even younger patients^16^,^17^. As symptoms of dilated cardiomyopathy appear and intensify in these patients over time, frequency and severity of arrhythmias similarly worsen and become life-threatening^18^. A strong negative correlation has been established between the occurrence of arrhythmias in DMD patients and associated prognosis^19^. As a result, patients are recommended against high-intensity adrenaline inducing activities beginning at early ages to avoid stressors that could exacerbate disease progression^14^. This evidence suggests that while left ventricular dilation and increased fibrosis contribute to the overall cardiac pathology, conduction abnormalities may be key factors in the onset and progression of DMD cardiomyopathy, leading to associated death^20^.

In the heart, the electrical conduction system is maintained by gap junction channels located at the intercalated discs of cardiomyocytes, allowing for proper signal propagation^21^. The most critical elements of this system are the connexin proteins, where six connexins assemble to form one hemichannel, and two hemichannels from neighboring myocytes join to form a gap junction. While multiple connexins exist in the heart, connexin43 (Cx43) is known as the major cardiac connexin, having the overall most abundant protein levels and being expressed throughout atrial and ventricular cardiomyocytes^21^,^22^,^23^. Interestingly, many cardiac diseases are marked by significant Cx43 remodeling in the form of “lateralization,” or redistribution to the lateral sides of cardiomyocyte membranes. This mislocalization allows for the opening of uncoupled hemichannels away from intercalated discs, leading to irregular electrical conduction and ion trafficking, contributing to the development of arrhythmias^24^,^25^,^26^.

As knowledge regarding the role of Cx43 and its structural properties has improved throughout the years, peptides mimetics have been designed to discriminate between hemichannels and gap junction channels for selective inhibition^27^. Representing one of the newer generation peptide mimetics, Gap26 corresponds to the first extracellular loop of Cx43. Due to the accessible location of this sequence, Gap26 is able to rapidly inhibit hemichannels within minutes. More recently, Gap19, corresponding to a nine amino acid sequence on the cytoplasmic loop of Cx43^28^, has been introduced as a more selective Cx43 inhibitor due to the sequence’s specific functional role. This peptide mimetic acts by blocking the interaction of the c-terminus with the cytoplasmic loop, a process required for the opening of hemichannels^21^,^27^,^29^. While this interaction also occurs in gap junctions, only hemichannels require the process for opening^21^, and thus are selectively inhibited while gap junctions remain functional^30^. Although Cx43 is also known to play a significant role in the brain, these peptide mimetics are unable to cross the blood-brain-barrier without an added TAT sequence, and thus limit the potential for off target effects away from the heart^30^.

Previous studies have shown the importance of Cx43 localization to the intercalated discs for overall cardiac health in various models of heart disease^26^. However, direct *in vivo* modulation of Cx43 has not been performed in general to assess anti-arrhythmogenic potential, and work done related to Cx43 in DMD is limited^31^. Here, we show that by utilizing mild mdx (dystrophin null) and severe mdx:utr (dystrophin and utrophin null) mouse models of DMD, as well as human DMD heart tissues, we observe an important role for Cx43 lateralization in DMD cardiac pathology. Also, by employing selective Cx43 peptide mimetics in isoproterenol (Iso) challenged DMD mice, as a model of cardiac stress, we find a potential route of therapeutic intervention to prevent the development of arrhythmias that normally occur^5^,^32^,^33^. By targeting Cx43 in this manner, we further observe a significant increase in overall survival of DMD mice. Our results demonstrate for the first time that selectively targeting Cx43 directly in DMD models prevents arrhythmogenesis and increases acute survival, while potentially improving long-term cardiac pathology and overall prognosis.

## Results

### Cx43 is lateralized in DMD mice, correlating with overall disease severity

**Figure 1:**
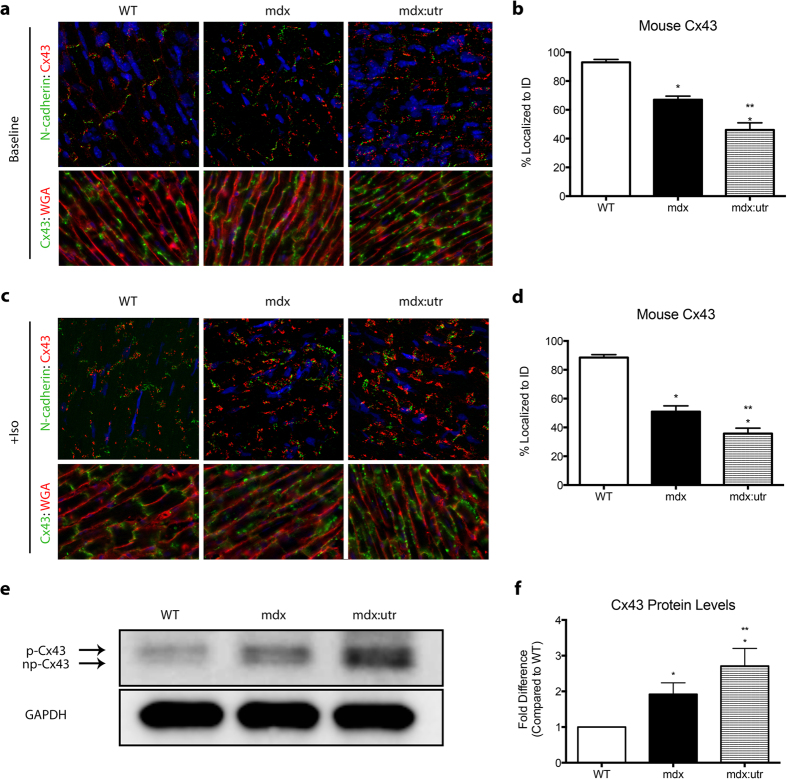
DMD mice show lateralized and upregulated Cx43 expression. (**a**) Representative immunofluorescence images of heart cryosections from 4 month old WT, mdx, and mdx:utr mice with antibodies reactive to Cx43 (red) and N-cadherin (green), as well as DAPI to stain nuclei (blue). (**b**) Quantification of baseline intercalated disc Cx43 expression, represented as percent of total Cx43 localized to intercalated discs (N = 5 (WT); 7 (mdx); 5 (mdx:utr). (**c**) Immunofluorescence images of heart cryosections from 4 month old Iso challenged (5 mg/kg IP) WT, mdx, and mdx:utr mice with antibodies as in (**a**). (**d**) Quantification of Iso challenged intercalated disc Cx43 expression, as represented in (**b**) (n = 6 (WT); 5 (mdx); 5 (mdx:utr). (**e**) Representative cropped western blot images of 4 month old WT, mdx, and mdx:utr heart protein homogenates probed for Cx43, with GAPDH as a loading control. Arrows indicate np-Cx43 or p-Cx43 bands, corresponding to differential phosphorylation states. Images of full-length blots are available as Supplemental Figure 3. (**f**) Quantification of (**e**), represented as fold difference compared to WT. * = p < 0.05 compared to WT, ** = p < 0.05 compared to mdx and WT.

The aberrant localization and protein expression of Cx43 have been largely associated with various heart disease models. While presence at the intercalated discs is required for proper cardiac conduction, lateralization and altered levels contribute significantly to cardiac pathology. To assess potential changes in expression and localization of Cx43 in the hearts of multiple models of DMD, we performed immunofluorescence (IF) staining on sections from WT, mdx, and mdx:utr mice at 2, 4, 8, and 12 months of age. mdx:utr mice were analyzed until the 4 month time point, as these animals typically only survive up to 3–4 months due to the severity of the disease^34^. In WT mice, Cx43 localizes tightly to the intercalated discs (93% ± 2% MEAN ± SEM, n = 5), critical for the formation of gap junctions and proper electrical signal conduction between cardiomyocytes (Fig. 1a,b). However, in mdx mouse hearts, even at 2, 4, and 8 months of age where no histopathology is present, Cx43 localization was significantly lateralized, with only 67% ± 3.5% (MEAN ± SEM, n = 7) expression at the intercalated discs (Fig. 1a,b). In addition, through analysis of the more severe mdx:utr double knock out model of DMD, an even lower proportion of Cx43 expression was observed at intercalated discs (46% ± 5% MEAN ± SEM, n = 5), demonstrating that while initial Cx43 remodeling occurs before overt cardiac pathology is present, it increases as signs of dilated cardiomyopathy and fibrosis appear (Fig. 1a,b). The mdx:utr model of DMD is known to develop significant cardiac inflammation, fibrosis, and degeneration, with related decreased overall cardiac function towards end stages of disease, similar to symptoms exhibited by patients^34^,^35^.

As Cx43 is known to have a very short half-life and undergo rapid turnover in response to cardiac stimuli^36^,^37^, we sought to examine the effect of β-adrenergic challenge on protein expression patterns. WT, mdx, and mdx:utr mice were challenged by Iso (5 mg/kg IP) and hearts were visualized for related changes in Cx43 within one hour (Fig. 1c). We found a significant increase in the lateralization of Cx43 in both mdx (51% ± 4% MEAN ± SEM, n = 5, at IDs) and mdx:utr (35.75% ± 3.75% MEAN ± SEM, n = 5, at IDs) mouse hearts, to levels greater than those seen prior to challenge, while WT hearts remained relatively unaffected (88% ± 2.5% MEAN ± SEM, n = 6) (Fig. 1c,d).

By performing western blot analysis on protein samples from each animal, we found that overall Cx43 levels were increased in DMD mice compared to WT (Fig. 1e,f). Specifically, mdx mice displayed a 1.92 ± 0.32 fold increase in Cx43 protein levels while the more severe mdx:utr mice exhibited even greater Cx43 upregulation (2.71 ± 0.49 fold), compared to WT mice (Fig. 1f). We further sought to examine whether the Pannexin-1 protein was dysregulated in DMD mice, as it has been shown to form hemichannels with properties similar to those formed by Cx43^38^. By western blot analysis of Panexin1, we found no significant differences in protein levels in mdx mice, compared to WT (Supplementary Fig. 1a,b). This indicated that Cx43 could specifically be playing a significant role in DMD pathological hearts in regards to channel function. Taken together, our findings show that Cx43 is not only redistributed to lateral sides of the cardiomyocytes but also upregulated at those positions.

### Human DMD hearts display lateralized and upregulated Cx43

**Figure 2:**
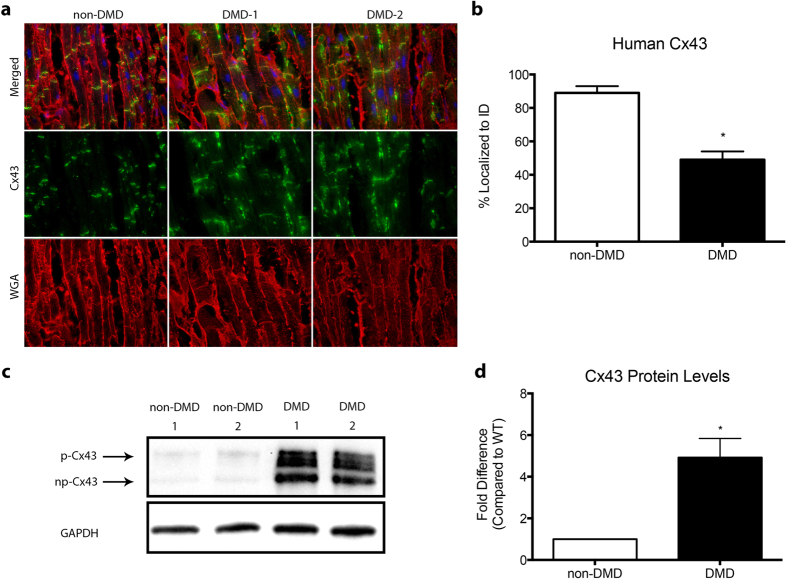
Human DMD hearts show altered Cx43 localization and expression. (**a**) Representative immunofluorescence images of heart cryosections from non-DMD and DMD patient hearts with antibodies reactive to Cx43 (green) and WGA (red), to demarcate cell borders, as well as DAPI to stain nuclei (blue). (**b**) Quantification of intercalated disc Cx43 expression, represented as percent of total Cx43 localized to intercalated discs with results from DMD1 and DMD2 combined for DMD. (**c**) Representative cropped western blot images of non-DMD and DMD patient heart protein homogenates probed for Cx43, with GAPDH as a loading control. Arrows indicate np and p bands of Cx43. Images of full-length blots are available as Supplemental Figure 3. (**d**) Quantification of (**c**), represented as fold difference of combined DMD1 and DMD2 results, represented as DMD, compared to non-DMD. * = p < 0.05 compared to non-DMD Human.

To examine whether altered Cx43 protein expression and lateralization are also present in human DMD, we performed Cx43 analysis on heart samples obtained from multiple non-DMD and DMD patients. Immunofluorescence indicates that DMD patients’ hearts exhibit significantly lateralized expression patterns of Cx43, compared to non-DMD individuals, at percentages similar to the severe DMD mouse model (49% ± 9% localized to IDs) (Fig. 2a,b).

Western blot analysis was performed on tissue samples from each patient analyzed by IF to quantify differences in protein expression levels. Results show that overall Cx43 is significantly upregulated in DMD patients compared to non-DMD (4.92 ± 0.92 fold), again at levels similar to, or even greater than, those seen in severe DMD mice (Fig. 2c,d). These findings indicate that Cx43 may play an important role in human DMD cardiomyopathy and that mouse models properly mimic levels and expression patterns seen in patients, even in DMD heart failure.

### mdx and mdx:utr models of DMD develop severe arrhythmias

**Figure 3:**
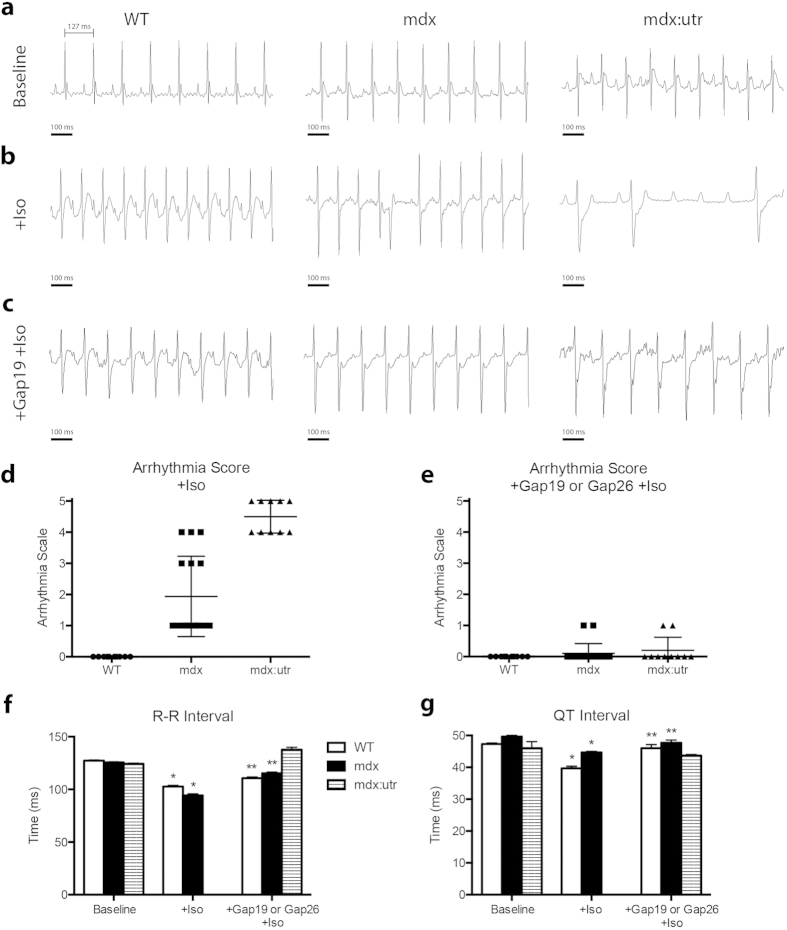
DMD mice develop severe arrhythmias upon Iso challenge that are prevented by Cx43 peptide mimetic treatment. (**a**) Representative baseline tracings of 4 month old WT, mdx, and mdx:utr whole animal ECGs following anesthesia. (**b**) ECG tracings after Iso challenge (5 mg/kg, IP). (**c**) Representative ECG tracings following treatment with Gap26 (1 μg/kg, IV) or Gap19 (10 μg/kg, IV) for 20 minutes and Iso challenge (5 mg/kg, IP). (**d-e**) Arrhythmia scores based on pre-determined scale where: 0 = no arrhythmias, 1 = single PVCs, 2 = double PVCs, 3 = triple PVCS or non-sustained VT, 4 = sustained VT or AV block, 5 = death. (**f-g**) Respective interval measurements made from randomly selected sequential beats of WT, mdx, and mdx:utr ECG tracings from baseline, +Iso challenge, and Cx43 peptide mimetic treated conditions. * = p < 0.05 compared to Baseline, ** = p < 0.05 compared to +Iso.

As proper protein levels and localization of Cx43 are known to be required for cardiac function, our initial findings led us to examine *in vivo* parameters that could be influenced by aberrant protein expression and localization of Cx43. WT, mdx, an mdx:utr mice underwent electrocardiography (ECG) to visualize disturbances in electrical conduction as a possible result of altered Cx43 activity. At baseline, no group showed significant spontaneous arrhythmias (Fig. 3a) and RR intervals did not show significant variation (WT: 127 ms; mdx: 126 ms; mdx:utr: 124 ms). Iso was used to challenge mice, at conditions equal to those previously mentioned, examining the susceptibility of these hearts to the development of conduction related disorders. Following administration, both mdx and mdx:utr mice developed arrhythmias within minutes, while WT mice did not show any signs of arrhythmias throughout the duration of analysis (Fig. 3b,d). Along with the initial occurrence of premature ventricular contractions (PVCs) in mdx and mdx:utr mice, R-R intervals were significantly decreased below 95 ms in each mouse (mdx: RR = 93 ms; mdx:utr: RR = 90 ms)(Fig. 3b) followed by undetermined values after arrhythmia scores worsened due to absent QRS complexes (Fig. 3f), translating to an increase in heart rate of about 165 beats per minute (bpm). WT mice also experienced shortened R-R intervals but to a lesser extent (WT: RR = 103 ms, Fig. 3f), increasing heart rate by about 100 bpm. While several mdx mice progressed to develop non-sustained ventricular tachycardia (VT) or AV blocks during the challenge (Arrhythmia Score = 2 or 4, accordingly), mdx:utr mice quickly advanced to sustained VT and AV block, and a majority died within 1 hour of initial Iso administration (Arrhythmia Score = 3, 4, or 5, accordingly) (Fig. 3d). ECG tracings also show a slight increase in QT intervals in mdx mice compared to WT both at baseline (WT = 47.3 ms, mdx = 49.7 ms) and after Iso (WT = 39.7 ms, mdx = 44.7 ms) (Fig. 3g). QT intervals could not be assessed properly in mdx:utr mice due to the rapid onset of VT and the related absence of distinct Q and T waves. As WT mice are known to be mainly unaffected by many fold higher doses of Iso^32^, these findings show a potential link between overall cardiac dysfunction in DMD and Cx43.

### Cx43 Inhibition Prevents Arrhythmias in DMD Models

As a possible mechanism to address the occurrence of arrhythmias in DMD mouse models, Cx43 hemichannels were directly targeted for functional inhibition using two different mimetic peptides, Gap26 and Gap19, designed to prevent the binding of sequences necessary for channel opening^27^. As Gap26 has been shown to be able to also inhibit gap junctions in a dose and time dependent manner, our studies utilized low, single doses. In addition, Gap19 was used as a more hemichannel selective peptide mimetic due to the role of the target sequence in hemichannel but not gap junction channel opening.

By treating mice with Gap 26 or Gap 19 prior to Iso administration, both DMD models showed significant decreases in conduction deficiencies (Fig. 3c). A majority of mdx mice (90%) were effectively protected from the development of VT and PVCs following even a low dose of Gap 26 (1 ug/kg) or Gap 19 (10 ug/kg) treatment (Arrhythmia Score = 0) (Fig. 3e). Optimal dosage was determined as lowest dose providing significant change in arrhythmia score. As Gap 26 corresponds to an extracellular sequence and Gap 19 corresponds to an intracellular sequence, a higher dose was required of Gap 19 to provide similar benefit as expected. Since the results of both treatments separately lead to equivalent scores and ECG patterns, measurements for Gap 26 and Gap 19 treated mice are expressed together. ECG intervals measured in treated, challenged DMD mice were significantly rescued to WT levels (WT: QT = 46 ms; mdx: QT = 47.7 ms; mdx:utr: QT = 44 ms) (Fig. 3g), with some intervals being further rescued even closer to baseline (mdx: RR = 115 ms compared to baseline mdx:126 ms) (Fig. 3f). To assess whether targeting Pannexin-1 could similarly protect DMD mice from challenge related symptoms, a Pannexin-1 inhibiting peptide mimetic was utilized. In both treated mdx and mdx:utr mice, arrhythmogenesis occurred following challenge to the same extent as untreated challenged mice (mdx: Arrhythmia Score = 3; mdx:utr: Arrhythmia Score = 5) (Supplementary Fig. 1c,d). In addition, western blot analysis was performed on baseline, Iso challenged, and Cx43 peptide mimetic treated Iso challenged cardiac samples to view a potential role of the sodium channel Na_v_1.5 in the rescue of lethality and arrhythmogenesis. Previous findings have shown the downregulation of Na_v_1.5 in mdx(5 cv) mice, and that transgenic upregulation provides a therapeutically beneficial restoration of sodium current that may prevent the development of arrhythmias^39^,^40^. Our results confirm that DMD mice display decreased protein levels of Na_v_1.5, but show that no significant changes occur following Cx43 peptide mimetic treatment (Supplementary Fig. 2a,b). Together, these findings indicate a significant, specific role for Cx43 hemichannel inhibition in preventing the onset of arrhythmias in DMD and in protecting DMD hearts from overall challenge-related effects.

### mdx mice treated with Gap 26 or Gap 19 survive following Iso challenge

**Figure 4:**
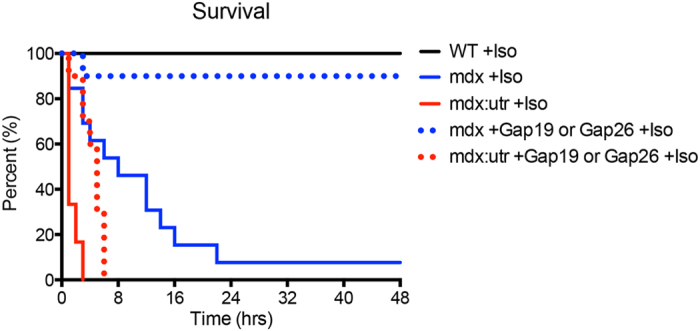
Cx43 peptide mimetic treatment protects DMD mice from death related to Iso challenge. Kaplan-Meier survival curve for WT, mdx, and mdx:utr Cx43 peptide mimetic treated or untreated, Iso challenged 4 month old mice. WT: +Iso n = 11; mdx: +Iso n = 15, +Gap19 or Gap26 +Iso n = 20; mdx:utr: +Iso n = 6, +Gap19 or Gap26 +Iso n = 10.

Following Iso challenge and the development of arrhythmias, we found that DMD mice displayed significant mortality within a short period of time^32^,^33^. A majority of mdx mice died within 24 hours after Iso was administered, while many mdx:utr mice died within only 1 hour following challenge (Fig. 4). However, 90% of mdx mice treated with either the Gap 26 or Gap 19 Cx43 peptide mimetic prior to Iso challenge were protected from both arrhythmogenesis and related lethality (Fig. 4). Although mdx:utr mice received substantial benefits from treatment in terms of ECG readouts, overall lethality was not prevented. These mice showed only slightly increased durations of survival (Fig. 4). Since mdx:utr mice do display overt cardiac and skeletal muscle histopathology as early as 2 months of age, models at this advanced stage of disease may not receive as significant a benefit as those in earlier stages such as mdx mice. To verify the selective benefits of Cx43 mediation, Pannexin-1 peptide mimetic treated DMD mice were challenged and monitored for survival. These mice died within durations comparable to untreated, isoproterenol challenged DMD mice (Supplementary Fig. 1e), consistent with observed arrhythmogenesis (Supplementary Fig. 1c,d) and an overall lack of Pannexin-1 peptide mimetic protective effects. Overall, our results show that mdx mice are successfully protected from arrhythmogenesis and death by Cx43 selective inhibition, and that while mdx:utr mice receive considerable cardiac benefits, treatment may be more effective if started at earlier ages before critical disease time points.

These results strongly implicate Cx43 in overall cardiac pathology and disease progression in DMD, and may have a significant impact on therapeutic development to combat a leading cause of death in the disease.

## Discussion

The identification of secondary factors for the treatment of DMD has become increasingly essential, as decades of research on dystrophin have demonstrated the difficulty in direct therapeutic targeting. In addition, the lack of sufficient knowledge related to the structural and functional roles of dystrophin in the heart have impeded cardiac research. More recently, as steroid therapy and ventilator use to combat respiratory failure became common practice, statistics have shown that heart failure has continued to grow as a prominent cause of death, with 100% of DMD patients over the age of 21 developing dilated cardiomyopathy^9^,^10^,^11^. With dilated cardiomyopathy characterized by arrhythmogenesis and protein remodeling, we sought to uncover downstream mechanisms in DMD that could be responsible for this pathology. Our studies focused on targeting Cx43, a gap junction protein and the most abundant cardiac connexin, required for maintaining proper ion trafficking and electrical signal propagation between cardiomyocytes. We discovered that through selective inhibition of Cx43 function, by administering peptide mimetics in a preemptive manner, we were able to prevent the occurrence of arrhythmias and lethality in Iso challenged mdx mice.

Although human DMD patients are known to develop dilated cardiomyopathy by late teens to early twenties, a majority of patients also develop arrhythmias at much younger ages. While these patients age and fibrosis occurs due to myocyte degeneration, the prevalence and intensity of arrhythmias increase accordingly. Our animal data shows that as early as 2 months of age, a low dose of Iso, administered as a minor cardiac stressor that is harmless to WT mice, is able to cause arrhythmogenesis in mild mdx mice. These arrhythmias are mainly constrained to PVCs, but as the animals age they become more intense. In addition, also at 2 months of age, the severe mdx:utr mouse shows that the same low dose of Iso causes significant ventricular arrhythmias leading to periods of tachycardia, bradycardia, and an eventual AV block indicative of heart failure. In both of these models, in addition to causing arrhythmias, the low dose of Iso is lethal, with mdx mice dying within an average of 24–48 hours and mdx:utr mice dying within an hour of monitoring.

Connexin remodeling has long been associated with cardiac disease, especially related to ischemia, hypertrophy, and heart failure. While typically Cx43 localizes to the intercalated discs of cardiomyocytes, forming gap junctions for the propagation of cardiac action potentials, a redistribution of the protein to the myocyte sarcolemma occurs in heart disease. In these positions, Cx43 proteins are believed to form nonjunctional hemichannels rather than functional gap junctions. These hemichannels may cause the influx of sodium and calcium ions, affecting membrane potential and impacting cardiac electrical conduction. This has the direct potential of leading to the development of cardiac arrhythmias. In addition, the exacerbated opening of Cx43 hemichannels can lead to the loss of the electrochemical gradient and intercellular metabolites, such as glutathione and ATP, making cardiomyocytes more susceptible to damage and volume overload^41^. Our data show that in both animal models and human DMD patients, Cx43 expression is dramatically altered, with a stark redistribution of the protein to the lateral sides of cardiomyocytes. Interestingly, while the mild mdx mouse shows a significant lateralization, the effect is even more pronounced in both the severe mdx:utr mouse and in human DMD patient hearts analyzed. Our results also show that protein levels are dramatically increased in all DMD tissues analyzed. Again, these results correspond to the degree of disease phenotype, where mdx:utr and human DMD patients demonstrated the greatest increases in protein levels. Results from both DMD1 and DMD2 tissues were very similar, indicating that Cx43 remodeling occurs in these patients independent of specific pathology, as the cause of death for DMD1 was heart failure and DMD2 was pulmonary embolism.

Cx43 localization is regulated through a variety of mechanisms, including protein trafficking following translation and overall stabilization of intercalated discs. In DMD, destabilization of the DGC includes the loss of anchored actin and desmin proteins. While both are known to play significant roles in maintaining cardiomyocyte morphology and function, recently the role of actin in trafficking of Cx43 has been specifically established. These studies show that disruptions in the interaction of Cx43 with the actin cytoskeleton lead to mislocalization of Cx43, especially in cardiomyopathy^42^. In addition, Cx43 is classically found by western blot in np-Cx43 and p-Cx43 bands, corresponding to non-phosphorylated and phosphorylated Cx43, respectively, due to relative amounts of phosphorylated residues affecting protein migration through the gel. Since at least 21 phosphorylation sites are known to exist on Cx43, with many required for protein trafficking, alterations in phosphorylation may also contribute to the lateralization of the protein and subsequent functional implications. Thus, these results merit further study.

While our results show that Cx43 is upregulated in all DMD models and human samples examined, Cx43 is typically downregulated in dilated cardiomyopathy and heart failure. As the DMD mouse and human hearts analyzed do not display cardiac hypertrophy, one of the conditions where this pattern is typically seen^26^, our findings demonstrate that in DMD cardiomyopathy Cx43 is remodeled in a novel manner. These findings represent the first time that Cx43 has been analyzed in the mdx:utr mouse model, form a direct correlation between the severity of DMD disease phenotype with Cx43, and describe a pattern of pathological Cx43 remodeling independent of hypertrophy specific to DMD.

Cx43 peptide mimetics have been used extensively in research related to ischemia/reperfusion, finding that when administered either before or during a period of ischemia, also at low doses, infarct size can be significantly reduced^43^. However, the effect of these peptides directly as anti-arrhythmic drugs has not been previously evaluated, especially in DMD. Our results demonstrate that DMD mice, which are susceptible to Iso-induced arrhythmias, are protected when either Gap26 or Gap19 is administered prior to challenge. As it has been shown that hemichannels can be targeted and inhibited in less than 15 minutes following peptide mimetic administration^44^, our challenge 20 minutes after treatment represents an effective and selective time point. Not only were arrhythmias prevented but RR and QT intervals were restored near WT levels. As a result, the beneficial effects of peptide mimetic treatment extend beyond anti-arrhythmic traits. We examined whether associated sodium channels could be implicated in overall cardiac benefits observed, especially because an increase in Na_v_1.5 through pharmacological intervention has been linked to a rescue in cardiac conduction^39^,^40^. However, western blotting showed that the effects of Cx43 peptides do not extend to Na_v_1.5, as protein levels in treated and untreated hearts were not significantly altered. In addition, although the challenge proved lethal for both mdx and mdx:utr mice within a short period of time, a majority of mdx mice that were treated prior to challenge survived the same dose of Iso. However, we also found that while mdx:utr mice did benefit from treatment in terms of ECG measurements, the challenge proved lethal within hours. As mdx:utr mice represent a severe model of DMD, and time points analyzed were following the development of overt pathological features such as cardiac fibrosis, cardiac functional deficits, and various skeletal muscle phenotypes that would be treated separately in patients^19^, we believe that these results show great potential for early intervention.

While our findings focus on the acute system, the use of Cx43 peptide mimetics as a preventative measure throughout a chronic disease could hold additional benefits, especially related to everyday tasks that would normally elicit a β-adrenergic response. As a result, studies related to long-term administration could prove significant. Together, we show that Cx43 peptide mimetics protect DMD mice from the development of arrhythmias, prevent lethality in mdx mice, and restore ECG intervals to WT measurements. As alternative Cx43 peptide mimetics have been previously shown to have favorable safety profiles in Phase I clinical trials for other indications, there is great potential for our results to have therapeutic implications in the rare disease of DMD.

## Methods

### Animals

Age matched WT, mdx, and mdx:utr male and female mice were analyzed at time points of 2, 4, 8, and 12 months. WT (C57BL/10J) and mdx (C57BL/10ScSn-Dmd^mdx^/J) mice were purchased from Jackson Laboratories. mdx:utr mice were generated by crossing mdx:utr(+/−) heterozygotes, a gift from Dr. Robert Grange (Virginia Tech), from a line originally derived and obtained from Drs. Mark Grady and Joshua Sanes (Washington University)^34^. Genotyping was performed using previously published sequences^45^. As mdx:utr mice on average do not survive later than 3–4 months of age, these mice were only analyzed up to the 4 month time point. No significant differences were found between sexes in remodeling or in response to peptide mimetic treatment. All animal experiments were approved by the IACUC of Rutgers New Jersey Medical School and performed in accordance with relevant guidelines and regulations.

### Human Samples

Two non-DMD and two DMD male human heart samples were obtained from the University of Maryland Brain and Tissue Bank, a member of the NIH NeuroBioBank network. All samples were dissected post-mortem. DMD1 cause of death was attributable to cardiac failure at age 15 while DMD2 cause of death was attributed to pulmonary thromboembolism at age 17. Informed consent was obtained from all subjects from whom tissues were analyzed. All human experiments were approved by the IRB of Rutgers University and performed in accordance with relevant guidelines and regulations.

### Immunofluorescence

Hearts from WT, mdx, and mdx:utr mice were dissected out, rinsed in sterile PBS, and frozen in O.C.T. using liquid nitrogen cooled isopentane. Human DMD heart samples were similarly frozen in O.C.T. using liquid nitrogen cooled isopentane. Cryosections were cut at 6 μm and stained with an antibody reactive to Cx43 (anti-mouse, Sigma, C8093, 1:500) along with a counterstain of wheat germ agglutinin (WGA, Invitrogen, 1:1000) to demarcate cell borders or N-cadherin (anti-rat DSHB, MNCD2, 1:20). Slides were coverslipped with a mounting medium containing DAPI and sections were imaged and analyzed using a Nikon A1R confocal (60 × objective, Fig. 1) or Eclipse T1 (40 × objective, Fig. 2) microscope and NIS-Elements AR software.

### Western Blotting

Hearts from WT, mdx, and mdx:utr mice were dissected out, rinsed in sterile PBS, and flash frozen in liquid nitrogen. Mouse tissues were homogenized in M-PER Mammalian Protein Extraction Reagent (Pierce) supplemented with EDTA, PMSF, NaVO_3_, Okadaic Acid, NaF, Benzamidine, and NaPyr. Human tissues were homogenized in RIPA buffer (50 mM Tris-HCl pH 7.4, 150 mM NaCl, 1% Nonidet P40, 0.5% Sodium Deoxycholate, 0.1% SDS, 5% glycerol) supplemented with protease and phosphatase inhibitor cocktails (Roche, cOmplete ULTRA Mini and PhosSTOP). Western blots performed without the use of proper phosphatase inhibitors yield a single band for Cx43 migrating near 43 kda, rather than separate np-Cx43 and p-Cx43 bands. Protein concentrations were estimated using a BCA kit (Pierce). 5 μg of protein per sample was separated on 12% gels (Bio-Rad) and run under the same experimental conditions, transferred to PVDF or nitrocellulose membranes, and probed with the following antibodies: Cx43 (Sigma, C8093, 1:8000), GAPDH (Cell Signaling, 3683, 1:2000), Panx1 (Santa Cruz, sc49695, 1:1000) or Na_v_1.5 (Sigma, S0819, 1:500). Blots were developed using enhanced chemiluminescence (ECL) and densitometric analysis was performed using Fuji Images or Bio-Rad Image Lab software.

### Electrocardiography

Whole animal electrocardiograms (ECGs) were recorded using needle electrodes in a Lead II configuration. Animals were anesthetized by Avertin (2,2,2-tribromoethanol, 290 mg/kg IP) and kept at a constant 37° temperature using a heating pad for the duration of analysis. ECG signals were acquired using a DSI Ponemah amplifier and pClamp Axoscope 10 software (Molecular Devices.) Following baseline reading, animals were treated for 20 minutes with the Connexin 43 mimetic peptide 43-Gap 26 (Anaspec, Sequence: VCYDKSFPISHVR, 1 ug/kg IV) or Gap 19 (Custom synthesized, Sequence: KQIEIKKFK, 10 ug/kg IV) via retro orbital injection. The sequence of Gap 26 corresponds to that of the first extracellular loop of Cx43 and inhibits the interaction between it and an adjacent Cx43 protein. The sequence of Gap 19 corresponds to that of the intracellular cytoplasmic loop of Cx43 and inhibits the interaction between it and the Cx43 C-terminus, which is required for hemichannel opening^21^,^27^,^28^. Negative control animals were treated for the same duration with a Pannexin-1 mimetic peptide (Anaspec, Sequence: WRQAAFVDSY, 10 ug/kg or 100 ug/kg IV) or a similar volume of saline. Following treatment time, mice were challenged by isoproterenol (Iso, Sigma, 5 mg/kg IP) and ECGs were constantly recorded. Arrhythmias were scored based on a point system where: 0 = no arrhythmias, 1 = single premature ventricular contractions (PVCs), 2 = double PVCs, 3 = non-sustained ventricular tachycardia (VT), 4 = sustained VT or atrioventricular (AV) block, and 5 = death. ECG parameters including RR, QRS, and QT intervals were measured at a significant number of randomly selected points to obtain representative values.

### Statistics

Data was evaluated for statistical significance using parametric analysis in GraphPad Prism or SigmaPlot software (ANOVA in the case of three groups or paired T-tests in the case of two groups) and expressed as mean ± SEM. P < 0.05 was considered statistically significant.

## Additional Information

**How to cite this article**: Gonzalez, J.P., *et al.* Selective Connexin43 Inhibition Prevents Isoproterenol-Induced Arrhythmias and Lethality in Muscular Dystrophy Mice. *Sci. Rep.* **5**, 13490; doi: 10.1038/srep13490 (2015).

## Supplementary Material

Supplementary Information
