# Erratum: Selective Connexin43 Inhibition Prevents Isoproterenol-Induced Arrhythmias and Lethality in Muscular Dystrophy Mice

**DOI:** 10.1038/srep15315

**Published:** 2015-10-20

**Authors:** J. Patrick Gonzalez, Jayalakshmi Ramachandran, Lai-Hua Xie, Jorge E. Contreras, Diego Fraidenraich

Scientific Reports 5: Article number: 13490; 10.1038/srep13490 published online: 08272015; updated: 10202015.

In the HTML version of this Article, Lai-Hua Xie is incorrectly listed as being affiliated with ‘Department of Pharmacology and Physiology, Rutgers Biomedical and Health Sciences, New Jersey Medical School, Newark, NJ USA’. The correct affiliation is listed below:

Department of Cell Biology and Molecular Medicine, Rutgers Biomedical and Health Sciences, New Jersey Medical School, Newark, NJ USA.

In addition, the Acknowledgements section is incomplete.

“We would like to thank Richard Gordan (Rutgers New Jersey Medical School) for his help establishing and interpreting ECGs”.

should read:

“We would like to thank Richard Gordan (Rutgers New Jersey Medical School) for his help establishing and interpreting ECGs, and Olmedo Restrepo (Rutgers New Jersey Medical School, Office of Special Programs) for his help with histology”.

