# Supplementary material for: Selective Connexin43 Inhibition Prevents Isoproterenol-Induced Arrhythmias and Lethality in Muscular Dystrophy Mice
Source: Sci Rep. 2015 Aug 27;5:13490. doi: 10.1038/srep13490 (PMC4550874; doi:10.1038/srep13490)
Supplement: Supplementary Information [file srep13490-s1.pdf]

Title

Selective Connexin43 Inhibition Prevents Isoproterenol-Induced Arrhythmias and Lethality in Muscular Dystrophy Mice

Authors

J. Patrick Gonzalez[1], Jayalakshmi Ramachandran[2], Jorge Contreras[2], Diego Fraidenraich[1]*

[1]Department of Cell Biology and Molecular Medicine, [2]Department of Pharmacology and Physiology, Rutgers Biomedical and Health Sciences, New Jersey Medical School, Newark, NJ

*To whom correspondence should be addressed: fraidedi@rutgers.edu

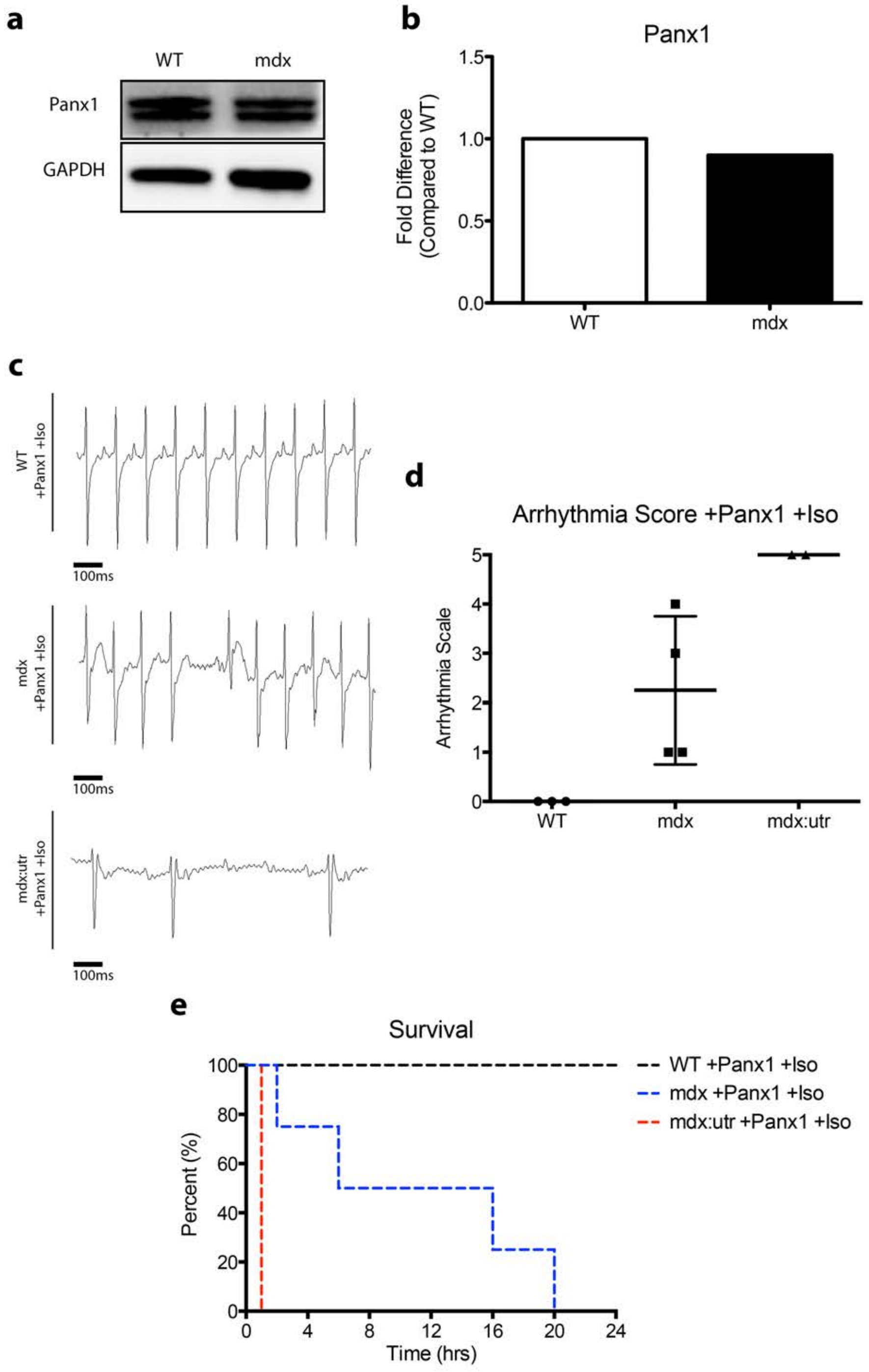

Supplementary Figure 1. Pannexin-1 levels and function are not implicated in DMD pathology. (**a**) Representative cropped western blot images from 4-month-old WT and mdx mouse heart protein homogenates probed for Pannexin-1 with GAPDH as a loading control. Images of full-length blots are available as Supplemental Figure 3. (**b**)

Quantification of (**a**), represented as fold difference compared to WT. (**c**) Representative ECG traces from Iso challenged WT, mdx, and mdx:utr mice treated with Panx1 peptide mimetic. Baseline and +Iso traces are depicted in Fig. 3a, b. (**d**) Arrhythmia scores for Panx1 treated, Iso challenged mice, using scoring system as described in Fig. 3. (**e**) Kaplan-Meier survival curve for Panx1 treated, Iso challenged WT (n=3), mdx (n=4), and mdx:utr (n=2) mice.

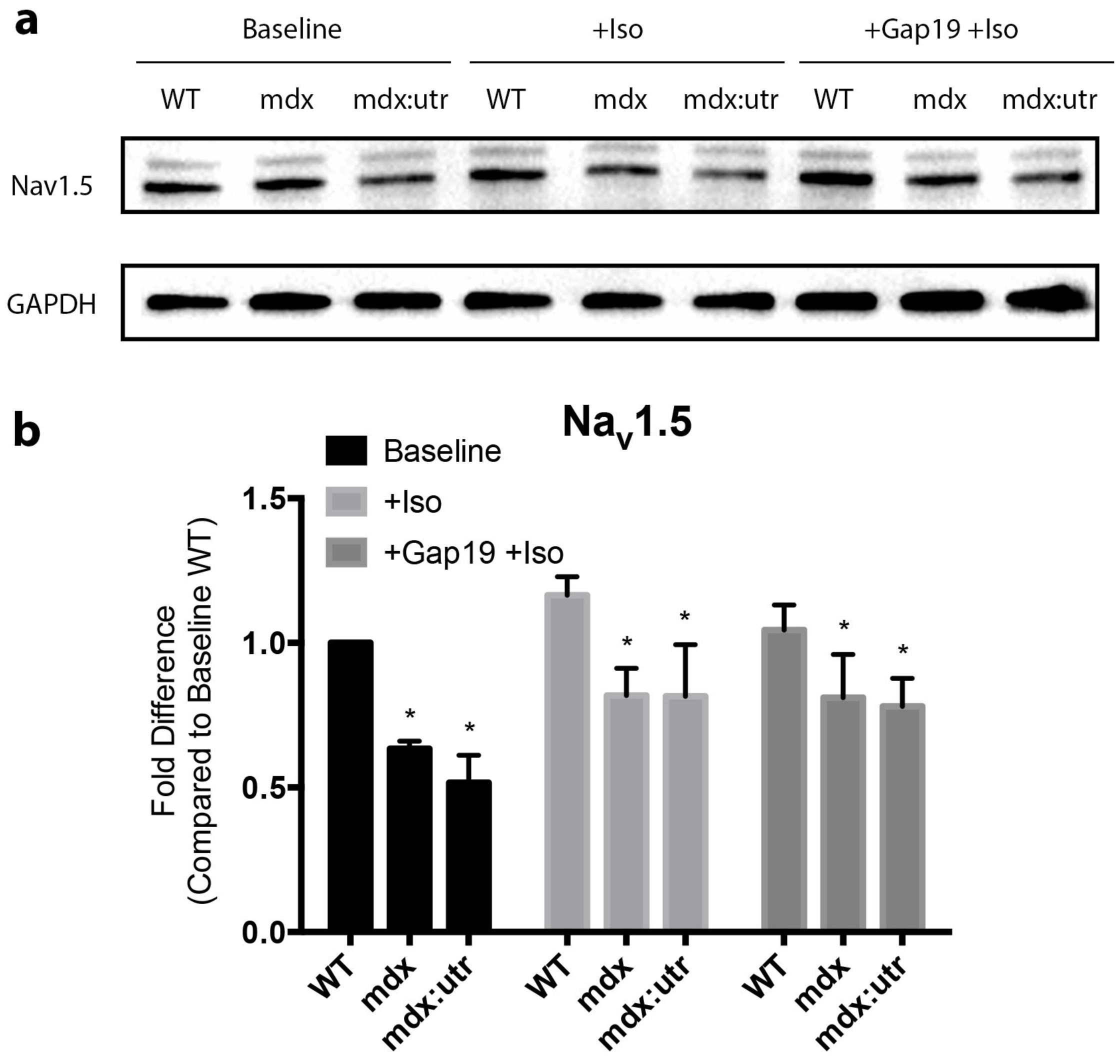

Supplementary Figure 2. $Na_v1.5$ protein levels are decreased in DMD but unchanged following Cx43 peptide mimetic treatment. (**a**) Representative cropped western blot images from 4-month old Baseline, Iso challenged, and Gap19 treated Iso challenged WT, mdx, and mdx:utr mice analyzing $Na_v1.5$ protein levels compared to GAPDH loading control. Images of full-length blots are available as Supplemental Figure 3. (**b**) Quantification of (**a**) , represented as fold difference compared to Baseline WT. *=P<0.05 compared to Baseline WT.

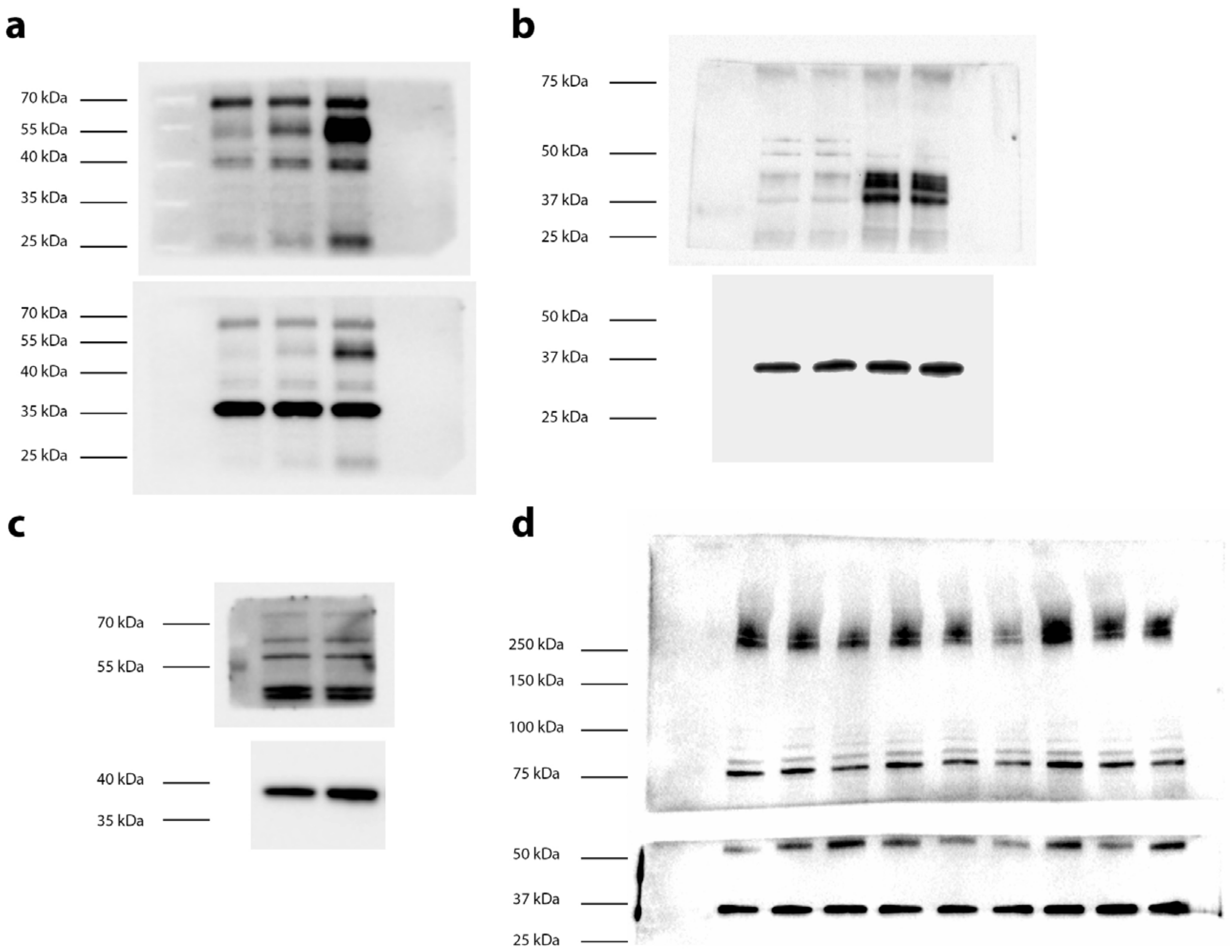

Supplementary Figure 3. Full-length western blots. Un-cropped images of (**a**) mouse Cx43 (top) and GAPDH (bottom) in Fig. 1e, (**b**) human Cx43 (top) and GAPDH (bottom) in Fig. 2c, (**c**) mouse Panx1 (top) and GAPDH (bottom) in Supplementary Fig. 1a, and (**d**) mouse $Na_v1.5$ (top) and GAPDH (bottom) in Supplementary Fig. 2a.
